# Introduction of Re(CO)_3_^+^/^99m^Tc(CO)_3_^+^ Organometallic Species into Vinylpyrrolidone-Allyliminodiacetate Copolymers

**DOI:** 10.3390/polym13111832

**Published:** 2021-06-01

**Authors:** Nikolay Ivanovich Gorshkov, Andrei Yur'evich Murko, Yulia Igorevna Zolotova, Olga Vladimirovna Nazarova, Valerii Dmitrievich Krasikov, Sergei Vasilievich Shatik, Evgenii Fedorovich Panarin

**Affiliations:** 1 Federal State Budgetary Institution of Science Institute of Macromolecular Compounds, Russian Academy of Sciences (IMC RAS), Russian Federation, V.O. Bolshoy pr. 31, 199004 Saint Petersburg, Russia; nitrobenzene@yandex.ru (A.Y.M.); nazaro@macro.spb.su (Y.I.Z.); nazaro@hq.macro.ru (O.V.N.); krasikov@lenchrom.ru (V.D.K.); panarin@hq.macro.ru (E.F.P.); 2 Federal State Budgetary Institution “Russian Research Center for Radiology and Surgical Technologies” of the Ministry of Health of the Russian Federation, Russian Federation, p. Pesochny, ul. Leningradskaya, 70, 197758 Saint Petersburg, Russia; shatik@hotmail.com

**Keywords:** vinylpyrrolidone-allylamine copolymers, iminodiacetate chelator, carbonyl, rhenium, technetium-99m

## Abstract

N-vinylpyrrolidone-co-allylamine copolymers (VP-co-AA) containing iminodiacetic (IDA) chelation units were prepared in the range of molecular masses of the copolymers from 9000 to 30,000 Da depending on polymerization conditions. Non-radioactive organometallic species Re(CO)_3_^+^ were introduced into polymeric carriers under mild conditions; the prepared metal–polymeric complexes were characterized by IR, NMR, ESI-MS and HPLC. IR spectra data confirmed the coordination of M(CO)_3_^+^ moiety to the polymeric backbone via IDA chelation unit (appearance of characteristic fac-M(CO)_3_^+^ vibrations (2005, 1890 cm^−1^), as well as the appearance of group of signals in ^1^H NMR spectra, corresponding to those inequivalent to methylene protons CH_2_COO (dd, 4.2 ppm), coordinated to metal ions. The optimal conditions for labeling the PVP-co-AA-IDA copolymers with radioactive ^99m^Tc(CO)_3_^+^ species were determined. The radiochemical yields reached 97%. The obtained radiolabeled polymers were stable in blood serum for 3 h. In vivo distribution experiments in intact animals showed the high primary accumulation of technetium-99m MPC (MM = 15,000 Da) in blood with subsequent excretion via the urinary tract.

## 1. Introduction

The chemistry of synthetic metal–polymer complexes (MPCs) is an urgent and vast area of polymer science. MPCs find wide application in various fields of science and engineering: the analysis and fixation of heavy metal ions, the design of biosensors, electro-optical devices, and antimicrobial, antibacterial, antiviral, antifungal, and antitumor activities [1,2,3]. MPCs are macromolecules containing metal ions, which, as a rule, are linked with polymer chains via chelation units [4,5]. In this respect, the use of metal–ligand interactions is versatile in this context, since the interaction strength and dynamic nature of the complexes can, in principle, be tuned by the variation of the metal ions, ligands, and linkers. Systems with various architectures can be formed, and, thus, polymers could be considered as irregulated macromolecular chelation systems [5].

The target delivery of radiolabeled compounds to specific sites of the human body is an important task of modern medicinal chemistry. Technetium-99m (^99m^Tc, t_1__/__2_ = 6 h, E = 141 keV) is the most commonly used radiometal for nuclear medical diagnostics [6,7,8]. In combination with 186 and 188 isotopes of its heavier homolog rhenium (186/188Re), technetium represents one of the first “theranostic pairs” for nuclear medical diagnosis and therapy [9]. The rhenium complex should be therapeutically active due to its structure, while the ^99m^Tc homologue allows for the visualization og drug pharmacokinetics. Proteins (e.g., monoclonal antibodies, MAb) have become indispensable tools for the target delivery of Tc/Re radioactive isotopes due to their high affinity for specific tumor sites. However, the synthesis and isolation of these macromolecules are extremely expensive and require complicated equipment and highly qualified personnel [10].

Another promising approach for the target delivery of radionuclides is the application of biologically active synthetic polymers (BASP). These objects are able to passively accumulate in target tumors via the enhanced permeation and retention (EPR) effect [11,12]. The EPR effect can be briefly described as follows. To supply a fast-growing tumor with blood, the vascularization of the tumor proceeds rapidly; as a result, the tumor blood vessels have an irregular structure. Their leaky architecture allows for the permeation of polymers into the cancerous tissue from the bloodstream (which is not possible in healthy blood vessels). Furthermore, usually there is no lymphatic drainage of a tumor (or it is disturbed), which prevents the rapid efflux of polymers from the tumor environment. As a result, concentrations of polymers in tumors may exceed their contents in healthy tissues by one to two orders of magnitude.

A large number of publications are devoted to introducing radionuclides into dendrimers, synthetic liposomes, micelles, hyperbranched and linear polymers, etc. [13,14,15,16,17,18]. The prospective carrier macromolecules should demonstrate a prolonged circulation time in blood, should have controlled dimensions (for effective localization in cells), and be easily cleared from an organism; accumulation in non-target organs in noticeable amounts is not acceptable. Flexible-chain synthetic water-soluble polymers are among the most promising objects for the targeted transport of radionuclides [13,16,17,18,19,20]. For instance, mannosylated dextrans grafted with diethylenetriaminepentaacetic acid (*DTPA*) chelation units and labeled with ^99m^Tc are approved for clinical use as agents for the visualization of defeated lymph nodes under the trade name “Lymphoseek”^TM^ [21].

The chemistry of N-vinylamide-based polymers (particularly, N-vinylpyrrolidone copolymers) has been comprehensively studied [22]; they are used in the world clinical practice as blood substituents and agents prolonging drug action [23].

During the last twenty years, organometallic species M(CO)_3_^+^ (M = ^99m^Tc, ^188,186^Re) have become very popular metal precursors for the labeling of biomolecules [24]. These organometallic fragments attract increasing interest due to their promising properties, such as relatively small size and low charge of metal atom (+1). The technetium(I) tricarbonyl complexes contain three carbon monoxides in facial coordination. The other three coordination sites are occupied by ligands with N, O, S, P, and C donor atoms. The complexes are obtained according to the two-step procedure, the first step being preparation of the “semiaqua” precursor fac-[^99m^Tc(H_2_O)_3_(CO)_3_]^+^ by reducing ^99m^TcO_4_^–^ ion with NaBH_4_ in the presence of CO gas or by reaction with reduction–carbonylation agent boranocarbonate [Na_2_BH_3_CO_2_] [25,26]. Then, the precursor reacts with ligands that replace labile water molecules. These complexes exhibit high in vivo stability, which make them an attractive platform for the development of new ^99m^Tc radiopharmaceuticals. The chemistry of the fac-[^99m^Tc(H_2_O)_3_(CO)_3_]^+^ complexes containing various chelating ligands has been studied extensively. Tridentate chelates are stable against substitution and thus show faster blood clearance than bidentate fac-[^99m^Tc(CO)_3_]^+^ complexes, which interact with blood proteins via the available coordination sites [27].

Natural macromolecules (antibodies and proteins), as well as BASP, have been successfully labeled with [^99m^Tc(CO)_3_]^+^ species; their biodistribution in laboratory animals was studied [28,29,30,31,32].

Previously, we have reported the introduction of the iminodiacetate (IDA) chelation unit into vinylpyrrolidone–vinylformamide copolymers; complexation of this macromolecular chelator with group (III) metals (gallium, indium) and the corresponding radionuclides (^68^Ga, ^111,113^In) has been investigated [33,34]. Iminodiacetate is also known as a powerful ligand for linking M(CO)_3_^+^ species in aqueous solutions [28].

In this work, we present the synthetic method for introducing the IDA chelation unit into copolymer carriers (vinylpyrrolidone-co-allylamine (VP-co-AA)). The synthesis of metal–polymeric complexes (MPCs) of the non-radioactive analog of ^99m^Tc (Re(CO)_3_) was carried out; structures of the products were studied, molecular masses and related characteristics were estimated; the technique for the chromatographic analysis of MPCs involving the use of monolithic sorbents was developed, and the procedure for labeling copolymers with radioactive ^99m^Tc(CO)_3_^+^ organometallic species was suggested.

## 2. Experimental

### 2.1. Materials

N-vinylpyrrolidone (*N*-VP, “Sigma-Aldrich”, St Louis, MO, USA), *N*-allylamine (*N*-VFA, Sigma-Aldrich, St Louis, MO, USA), and 2,2′-azobisisobutyronitrile initiator (AIBN, Biolar, high purity grade, St Petersburg, Russian Federation) were used in copolymerization. The solvents and reactants (reagent and analytical purity grades) were purchased from Vekton (St Petersburg, Russian Federation) and Sigma-Aldrich (St Louis, MO, USA).

*N*-VP and *N*-AA monomers were purified by distillation under vacuum (b.p. = 69 °C (3 mm Hg), n_D_^20^ = 1.5120; b.p. = 65 °C (4 mm Hg), n_D_^20^ = 1.4920, respectively). AIBN was purified by recrystallization from an ethanol:chloroform mixture (3:1), m.p. = 103 °C.

### 2.2. Instruments and Measurements

^1^H and ^13^C NMR spectra were recorded using a Bruker Avance II-500 WB spectrometer (Billerica, USA) in deuterated solvent (D_2_O) purchased from Sigma-Aldrich, (St Louis, MO, USA). Chemical shifts were referenced to the signal of residual non-deuterated solvent (water, 4.8 ppm) and the signal of external tetramethylsilane standard.

IR spectra were obtained using a Shimadzu Prestige FTIR spectrometer (Kyoto, Japan) in KBr pellets.

UV spectra were registered using a Shimadzu UV-1280 (Kyoto, Japan) spectrophotometer.

Mass spectra were obtained with a Bruker APEX-Qe ESI FT-ICR instrument (Billerica, MA, USA).

Chromatographic analysis was performed with the use of a Smartline HPLC instrument (Knauer, Geretsried, Germany) equipped with a JetStream column thermostat, refractometric and spectrophotometric detectors. The registration of chromatograms and calculations of molecular masses and other parameters were performed using ClarityChrom GPC/SEC V.2.6 xx setup (Geretsried, Germany). An Ultrahydrogel linear SEC column (7.8 × 300 mm) with a pre-column (0.6 × 40 mm, Waters, Milford, MS, USA) was used for the analysis of the copolymers. Chromatographic analyses were carried out in aqueous solution of 0.2 M NaCl at 25 °C. Calibration dependences for the columns were plotted using the data for the previously characterized poly(*N*-vinylformamide) standards in 0.2 M aqueous solution of NaCl; the values of the Kuhn–Mark–Houwink constants were K = 10.74 × 10^–3^ and α = 0.76 ± 0.04 [35]. Ultrashort monolith CIM^TM^ (Convection Interaction Media) columns (CIM disks, 1.2 × 0.5 cm) (Ajdovščina, Slovenia) were used for monitoring the polymer–metal interaction and for the evaluation of radiochemical yields.

Intrinsic viscosity [η] was measured using an Ubbelohde viscometer, (Vekton, St Petersburg, Russia). Relative viscosity (η_r_) was calculated as an initial slope of the ln(η_r_) = f(c) dependence, i.e., in the region where η_r_ is the relative viscosity of a solution at concentration *c*. The measurements were performed in 0.1 M solution of sodium acetate at 25 °C.

The ^99m^Tc eluate was purchased from the Khlopin Radium Institute (St Petersburg, Russia). Radioactivity was measured using a Curie mentor 3/4 dose calibrator and a GabiStar specialized chromatographic flow detector (Raytest, Straubenhardt, Germany).

### 2.3. Synthesis of Copolymers

#### 2.3.1. Synthesis of VP-co-AIDA

Reactions of free radical copolymerization of VP and AA were carried out in sealed ampoules in argon atmosphere at 60 °C in ethanol or water solutions for 48 h. Concentrations of monomers were 50 wt.%, and the concentration of the initiator (2,2-azobisisobutironitrile, AIBN) was 1 wt.% with respect to monomer mass. The polymers obtained in ethanol solution were precipitated into diethyl ether and dialyzed through a 1000-MWCO (molecular weight cutoff) dialysis tubing (Spectra/Por 7, New Brunswick, NJ, USA) against 2% NaCl water solution for 24 h and against water for 24 h. Then, the copolymers were lyophilized. The copolymers synthesized in water solutions were dialyzed and lyophilized.

Compositions of the copolymers were determined by two methods: (1) using the absorption spectra of complexes of AA units and 2,4,6,-trinitrobenzenesulfonic acid, *λ*_max_ = 420 nm, (2) by potentiometric titration of AA units with 0.1 N HCl solution. Molecular masses (MMs) of copolymers were estimated viscometrically using the Mark–Kuhn–Houwink parameters found for PVP. The values of intrinsic viscosity (η) were determined in 0.1 M Na_2_SO_4_ solution (25 °C).

**Scheme 1:**
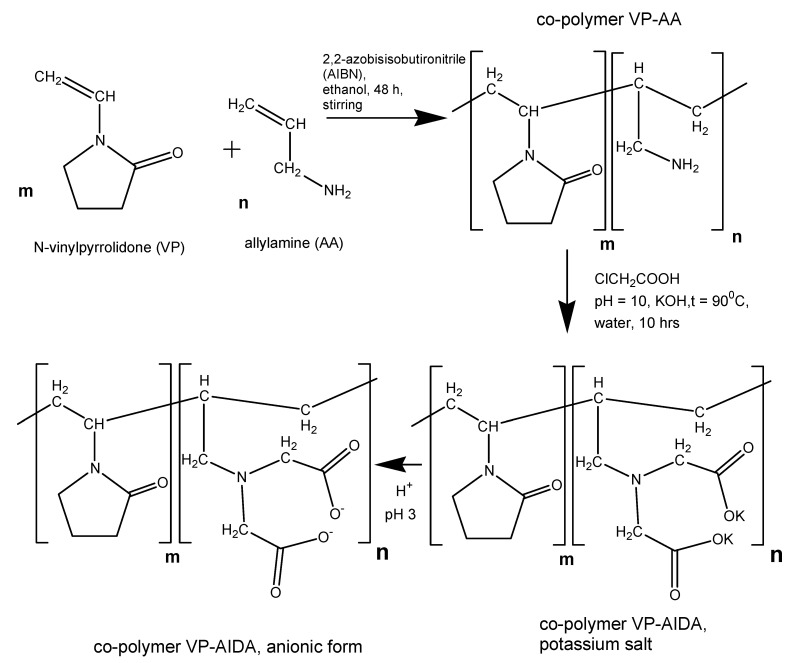
Synthesis of VP-co-AIDA copolymers.

The reaction between polymer amino groups and monochloroacetic acid was carried out in KOH water solution (pH = 10) at 90 °C [34,36]. The content of IDA-containing groups was estimated by potentiometric titration. The reaction schemes, monomer ratios, intrinsic viscosities, and SEC data are presented in Scheme 1 and in Table 1 and Table 2.

Analytical data for VP-co-AIDA:

Sample with MM 9000 Da

IR (KBr pellets): 1645 (w) (COO), 1590 (vs.)(N-C=O),

^1^H NMR (D_2_O): 3.75 s (H3), 3.62 s (H4), 3.27 s (H5), 3.10 s (H3) (lactone ring), 2.89 m (H3) [AA], 2.41 s (H1), 2.28 (H2), 1.99 s (H1′), 1.92 s (H2′) (aliphatic backbone)

ESI MS, *m*/*z* (10+), 1000 (broad)

Sample with MM 15,000 Da

IR (KBr pellets): 1645 (w) (COO), 1590 (vs.)(N-C=O),

^1^H NMR (D_2_O): 3.75 s (H3), 3.62 s (H4), 3.27 s (H5), 3.10 s (H3) (lactone ring), 2.89 m (H3) [AA], 2.41 s (H1), 2.28 (H2), 1.99 s (H1′), 1.92 s (H2′) (aliphatic backbone)

ESI MS, *m*/*z* (10+), 1000 (broad)

Sample with MM 15,000 Da

IR (KBr pellets): 1645 (w) (COO), 1590 (vs.)(N-C=O),

^1^H NMR (D_2_O): 3.75 s (H3), 3.62 s (H4), 3.27 s (H5), 3.10 s (H3) (lactone ring), 2.89 m (H3) [AA], 2.41 s (H1), 2.28 (H2), 1.99 s (H1′), 1.92 s (H2′) (aliphatic backbone)

ESI MS, *m*/*z* (10+), 1500 (broad)

Sample with MM 30,000 Da

IR (KBr pellets): 1645 (w) (COO), 1590 (vs.)(N-C=O),

^1^H NMR (D_2_O): 3.75 s (H3), 3.62 s (H4), 3.27 s (H5), 3.10 s (H3) (lactone ring), 2.89 m (H3) [AA], 2.41 s (H1), 2.28 (H2), 1.99 s (H1′), 1.92 s (H2′) (aliphatic backbone)

ESI MS, *m*/*z* (10+), 2900 (broad)

#### 2.3.2. Synthesis of VP-co-AIDA-Re(CO)_3_ Metal-Polymer Complexes

The aqueous solution of [Re(CO)_3_(H_2_O)_3_]^+^Cl was prepared by boiling Re(CO)_5_Cl (100 mg) in 5 mL of water for 48 h. VP-co-AA-IDA samples (MM = 15 and 30 kDa) were dissolved in 3 mL of water, and equimolar amounts of aqueous solution of [Re(CO)_3_(H_2_O)_3_]^+^Cl were added. The pH value of reaction mixture was maintained at 6 in order to avoid the formation of hydrolyzed rhenium carbonyl species. The mixture was heated for 3–4 h at 70 °C or kept for 48 h at room temperature. The reaction was followed by ultra-fast HPLC on carboxymethyl (CM) monolith columns in the isocratic elution mode (0.1% TFA-methanol 50/50, 0.3 mL/min, UV detection (λ_n_ = 210, 254, 320 360 nm) until the conversion of organometallic precursor was complete. Then, the reaction mixture was purified via membrane dialysis against water for 24 h and lyophilized.

Analytical data for sample with MM ≈ 15,000 Da:

UV (water): 230 nm

IR (KBr), cm^−1^: 2005 (vs), 1890 (vs) (fac-Re(CO)_3_), 1591 (vs) (N-C=O (lactone ring))

ESI MS: *m*/*z* 10^+^ 1500 (broad)

^1^H NMR (D_2_O), ppm: 3.75 s (H3), 3.62 s (H4), 3.27 s (H5), 3.10 s (H3) (lactone ring), 3.325, 3.281, 3.231, 3.186. (dd, AIDA) 2.89 m (H3) [AA], 2.41 s (H1), 2.28 (H2), 1.99 s (H1′), 1.92 s (H2′) (aliphatic backbone).

#### 2.3.3. Radiochemical Synthesis of VP-co-AA-IDA- ^99m^Tc(CO)_3_ and Evaluation of In Vitro Stability

The organometallic precursor [^99m^Tc (CO)_3_(H_2_O)_3_]^+^ was prepared by reducing ^99m^TcO_4_^−^ eluate (T_1/2_ = 6 hrs, E_γ_ = 140 keV) (1 mL, average activity: 1–2 GBq) with Na_2_BH_3_CO_2_ in the presence of sodium–potassium tartrate at 90 °C for 10 min according to the procedure described elsewhere [37,38]. Then, the reaction mixture was neutralized to pH = 6.5 with phosphate buffer solution.

An amount of 0.5 mL of aqueous solution of the copolymer (C = 10^−3^–10^−4^ M, pH 1–8) was added to 10 µL of prepared solution of [^99m^Tc (CO)_3_(H_2_O)_3_]^+^, (activity 0.01–0.02 GBq), and the mixture was heated at 70 °C for 5–20 min. The reaction was monitored by HPLC on ultra-short CIM CM columns in the isocratic mode, elution system: 0.1% aqueous TFA, 0.3 mL/min, detection: UV (λ = 210 nm) and a gamma-radioactivity detector. The maximal achieved radiochemical yield was 95%.

The in vitro stability of VP-co-AA-IDA- ^99m^Tc(CO)_3_ (MM 30 kDa) was evaluated as follows. An amount of 150 µL of solution of rat blood serum in 0.1 M NaCl was added to 50 µL of solution of ^99m^Tc-labeled copolymer in phosphate buffer (pH = 7.4). The mixture was incubated at 37 °C for 15–180 min. The stability of the labeled copolymer in blood serum was estimated by HPLC on Ultrahydrogel Linear SEC column (0.78 × 30 cm), elution: 0.2 M NaCl, 0.8 mL/min.

#### 2.3.4. Biodistribution Studies

The procedure was as described previously [34].

The biodistribution of the MPC in organisms of linear intact laboratory Wistar rats (body weight about 200 g) was studied according to the following protocol. A ^99m^Tc(CO)_3_-VP-AIDA MPC(15 kDa) (1–3 MBq, 0.25 mL), adjusted to pH 7.4 with phosphate buffer system, was injected into the caudal vein. After injection, the animals were sacrificed by decapitation. The target organs, blood (0.5 g), and tissues samples (0.1–0.6 g) and blood samples were placed in tubes with the same geometry. The radioactivity measurements of the ^68^Ga were performed using a calibrated gamma meter by direct radiometry. The accumulation of the MPC in organs and tissues was calculated as a percentage of the total introduced activity per 1 g of an organ/tissue. Since the ^99m^Tc radioisotope is short-lived (the half-life is 6 h, which is comparable with the time of experiment), this factor was taken into account when recalculating the accumulation in organs and tissues. Prior to measuring the radioactivity of a series of organs and tissues, the radioactivity of the reference sample of the same MPC was measured in the tube of the same geometry. The measurement time of the standard sample and selected organs and issue was equal. The measured radioactivity was accepted as the total activity for a specific series, taking into account measurement time.

## 3. Results and Discussion

Copolymers of N-vinylpyrrolidone were selected as carriers for metal radionuclides due to their biocompatibility and cell membrane permeation properties [12]. It is known that molecular masses of polymers used in pharmaceutical preparations should not exceed 40–50 kDa, and their hydrodynamic radii should lie in the range of 5–10 nm to permit renal or hepatobiliary excretion [11,12]. Thus, the directional design of copolymers with the desired chemical and structural characteristics (MM, polydispersity, hydrodynamic properties) is important for the target transport of radionuclides.

It should be noted that lactone rings of poly-N-vinylpyrrolidone (PVP) homopolymer contain donor atoms (N, O), which potentially can bind metal ions. Therefore, firstly, we studied the possibility of complexation between Re(CO)_3_^+^ organometallic compound and PVP (MM = 30 kDa) by HPLC on ultrashort monolith columns (carboxymethyl modification). The use of monolithic sorbents, which are able to separate both homopolymers in a gradient mode and metal ions [33,34], may be a possible solution of a complex problem of separation of flexible-chain BASP and metal ions.

Monitoring the reaction at various pH values and temperatures demonstrated the absence of any noticeable coordination activity of PVP.

In order to impart complexation ability to PVP, its copolymers with directionally attached iminodiacetic (IDA) chelating units were synthesized. The synthetic pathway is presented in Scheme 1.

Copolymers of VP with AA were obtained by radical copolymerization in ethanol solution (where the total concentration of monomers was equal to 50 wt.%) using azobisisobutyronitrile (AIBN, 1 wt.%) as an initiator. It is known that AA shows relatively low reactivity in copolymerization with VP [36], and an increase in the AA content in the monomer mixture leads to a decrease in MM of the resulting copolymer. Thus, we synthesized VP–AA copolymers with contents of AA units varying from 6 to 12 mol.%; their Mη ranged from 9000 to 30,000 Da (Table 2), which meets the requirements for polymer radiolabeling and target drug transport.

IDA chelation groups were introduced into the polymeric backbone by the alkylation of amino groups of the VP–AA copolymer with monochloracetic acid (Scheme 1).

Contents of amino groups in the resulting VP–AA samples were determined by UV spectroscopy at the wavelength of 420 nm (which is characteristic for complexes between primary amines and 2,4,6-trinitrobenzenesulfonic acid). Contents of carboxylic groups in VP-co-AIDA copolymers were evaluated by potentiometric titration of a sample with 0.1 N solution of HCl. MM of copolymers were calculated using the SEC data (Table 1) (eluent: 0.1 M aqueous solution of NaCl, 25 °C). The calibration dependence was plotted using Mark–Kuhn–Houwink constants for poly-N-vinylformamide [35].

As can be seen, no significant changes in MWD between starting co-polymers and resulting MPCs were observed. The same tendency was observed previously [33,34] using more precise methods, e.g., molecular hydrodynamics and optics in combination with SEC. This fact suggests the hypothesis, that the introduction of metals into the macromolecular matrix will not significantly disturb its hydrodynamical and biological properties and, thus, should not drastically disturb its biological behavior.

Rhenium was chosen for the experiments, since it is a closest chemical analogue of technetium. In order to develop the technique for the radiochemical preparation of MPC at the non-carrier added level (where metal ion concentration is equal to 10^−10^–10^−13^ M), it is necessary to optimize synthetic conditions for the procedures involving noticeable quantities of the polymer carrier and metal ions, to determine the composition and structure of the target product and to optimize chromatographic techniques. The latter is especially important, since chromatography is the main method for the determination of radiochemical yields of radiolabeled compounds. The reaction between organometallic [Re(CO)_3_(H_2_O)_3_]^+^ precursor and VP-co-AA-IDA was carried out at room temperature for 48 h or at 70 °C for 4 h.

Reaction kinetics was monitored by HPLC (the yield of the metal–polymer complex was estimated). In order to reduce the time of analysis, monolith ultra-short columns were used. Due to unusual the pore structure and small length (0.3 cm) of monolith columns, they make it possible to perform fast and effective separations of high and low molecular weight compounds (5–10 min). The latter advantage is critical for radiochemical synthesis, since diagnostic radionuclides have short half-lives. However, CIM monolith columns cannot separate polymers according to their molecular masses, because the sorption mechanism is realized in the column during the chromatographic experiment (i.e., a polymer is adsorbed on the matrix).

Various derivatives of the basic material (glycidyl methacrylate/ethylenediamine dimethacrylate, GMA-EDMA), including strong cation and anion exchangers (DEAE, quaternary amine, SO_3_, carboxymethyl) were tested. Strange as it may seem, the carboxymethyl (CM) stationary phase was found to be the most effective in separation of a macromolecular component from an organometallic moiety in organic–aquatic media.

**Figure 1:**
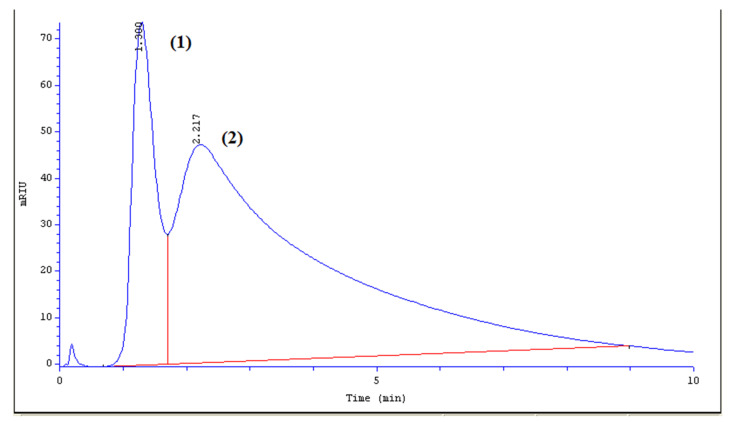
HPLC profile of reaction mixture (CIM-CM column, 1 × 0.5 cm) containing VP-co-AIDA copolymer and Re(CO)_3_(H_2_O)_3_. Elution system: 0.1% TFA/methanol 50:50; isocratic elution; UV detection (λ = 360 nm); RI detection; flow rate: 0.3 mL/min. The sharp peak (**1**) corresponds to metal–polymer complex VP-co-AIDA-Re(CO)_3_; the broad peak (**2**) is attributed to Re(CO)_3_(H_2_O)_3_.

A typical HPLC profile of the reaction mixture [Re(CO_3_)]^+^ + VP-co-AIDA (MM ≈ 15 kDa) is presented in Figure 1. It should be noted that the UV spectrum of the starting copolymer includes the characteristic peak at a wavelength of 210 nm. An additional absorption maximum at 360 nm is present in the UV spectra of the resulting MPC, as well as in the spectra of aqueous [Re(CO)_3_(H_2_O)_3_]^+^. The appearance of absorbance at this wavelength in the HPLC profile of the macromolecular component clearly indicates the formation of MPC.

The retention time (RT) of macromolecular components is close to the free volume of the column, while the peak of [Re(CO)_3_(H_2_O)_3_]^+^ complex has a higher RT. The shape of wide peak (2) can be explained by the rapid exchange of labile water molecules in the coordination spheres of [Re(CO)_3_(H_2_O)_3_]^+^ particles and the sorption of these particles on the surface of the sorbent.

**Figure 2:**
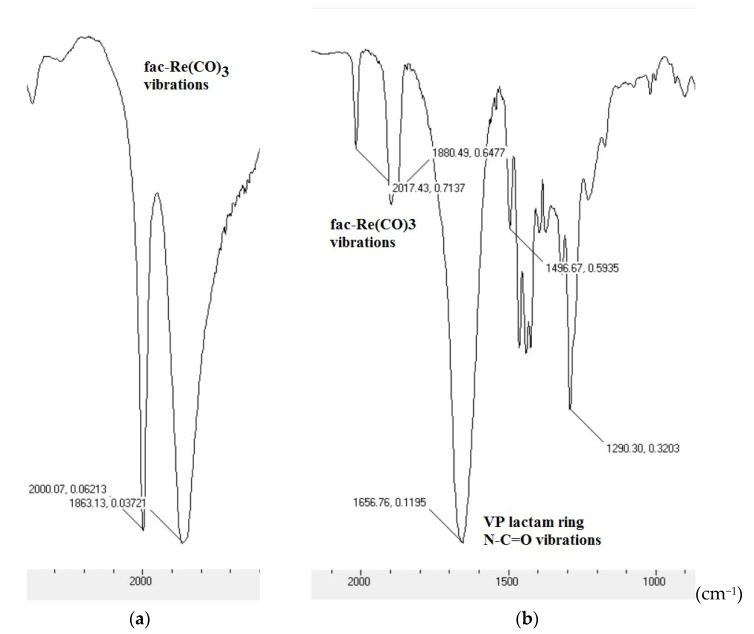
IR spectra of reference [Re(CO)_3_Br_3_][N(Et)_4_]_2_ (**a**) VP-co-AIDA-Re(CO)_3_ (15,000 Da) (**b**), KBr pellets.

The reaction mixture was purified by dialysis against water, and the target MPC was freeze-dried. Coordination between [Re(CO_3_)]^+^ and VP-co-AIDA was confirmed by IR spectroscopy (strong characteristic bands of fac-[Re(CO_3_)]^+^ moiety (2017, 1880 cm^−1^) was appeared and significantly shifted in contrast to reference [Re(CO)_3_Br_3_][N(Et)_4_]_2_ (2000, 1863 cm^−1^) (Figure 2)).

^1^H NMR spectra of VP-AIDA copolymers is practically the same as PVP, because in general, the most intensive signals are attributed to the PVP backbone. Signals of AIDA protons are underplayed with more intense peaks of the PVP backbone. The same effect was observed earlier in the case of analogous polymers VP-VIDA [27].

However, in the ^1^H NMR spectra of metal–polymer complexes, downfield shifts of the signals attributed to AIDA methylene protons (2.89 ppm, (m)) linked to the polymeric backbone were observed. In addition, the fine structure of the signals of methylene N-CH_2_-CO_2_ protons (dd, 3. 4 ppm)) becomes inequivalent upon coordination to metal core. The same effect occurring in ^1^H NMR spectra of similar metal–polymer complexes Ga-VP-VIDA has been previously described [27]. The coupling of signals of methylene protons was also observed in ^1^H NMR spectra of low molecular weight complex [Re(CO)_3_(IDA)][N(Et)_4_] [34].

**Figure 3:**
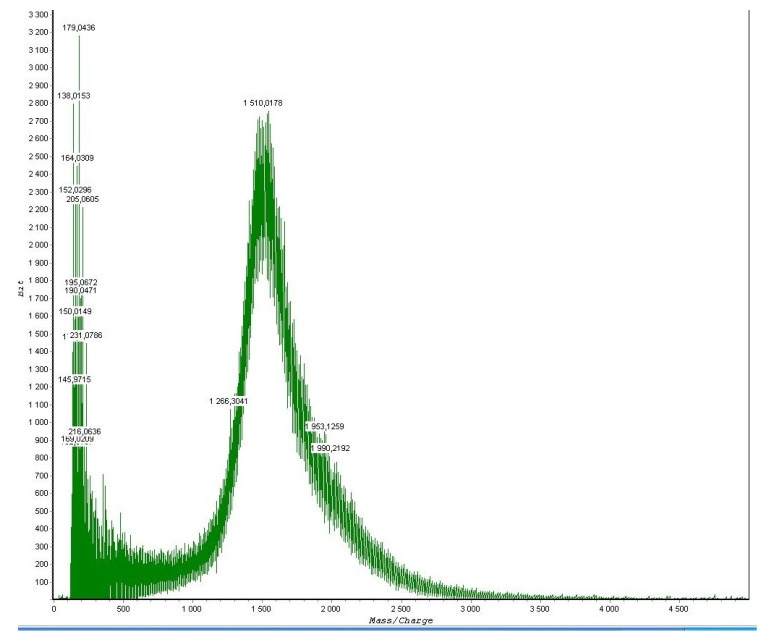
ESI-MS data for the VP-co-AIDA-Re(CO)_3_ (MM 15,000 Da).

The MM of polymers and MPC were determined by electrospray mass-spectrometry (ESI MS). The unique ability of ESI to produce multiply charged ions extends this extremely soft technique to a higher mass range, even for analyzers with a limited mass range. Unfortunately, even polymers with low polydispersities and high masses give very complex mass spectra due to multiple charging [39,40]. Although mass spectrometry gives the exact molecular weights of individual molecules, accurate molecular weight distributions (MWD) of synthetic polymers can only be obtained in some instances. This is because samples of synthetic polymers do not have uniform chain lengths, but demonstrate a distribution of molecular weights. A representative ESI mass-spectrum of VP-co-AIDA-[Re(CO_3_)] metal–polymer complex is presented in Figure 3. It should be noted that the shape of ESI MS mass distribution generally resembles the SEC profile of a sample. The overall charge of macromolecular fractions is 10^+^, and this value does not depend on MM; besides, the introduction of Re(CO_3_) does not have any significant influence on mass distribution.

**Figure 4:**
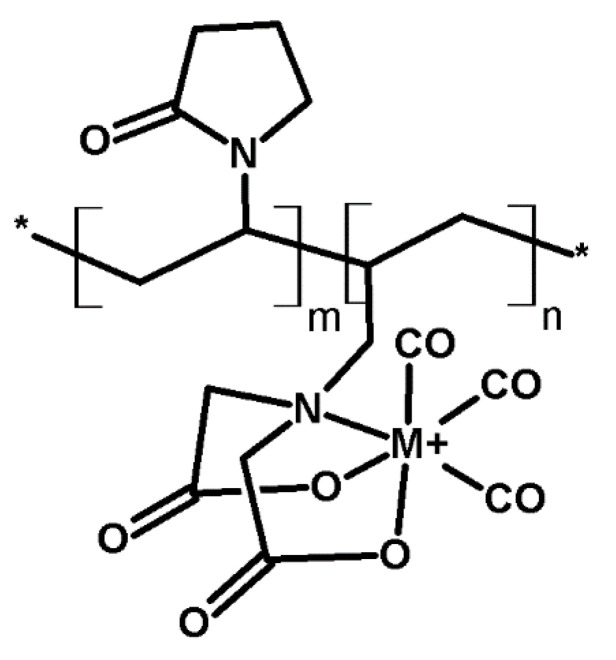
Proposed structure of VP-co-AIDA-Re(CO)_3_ MPC. Analogous structure was estimated by XRD studies for low-molecular complex [Re(CO)_3_(IDA)][N(Et)_4_] [34].

Thus, the proposed structure of the obtained MPCs could be presented as shown at Figure 4.

The main goals of the present work were to develop an effective procedure for the radiolabeling of VP-co-AA-IDA with radioactive ^99m^Tc(CO)_3_^+^ moiety, and to achieve high radiochemical yields and high degrees of purity necessary for biodistridution studies. It is known that the reactivity of Tc carbonyls is higher than that of Re analogs [24]. The following optimal conditions for the radiolabeling reaction were found: 80 °C, 20 min, and the copolymer concentration equal to 10^−3^ M.

**Figure 5:**
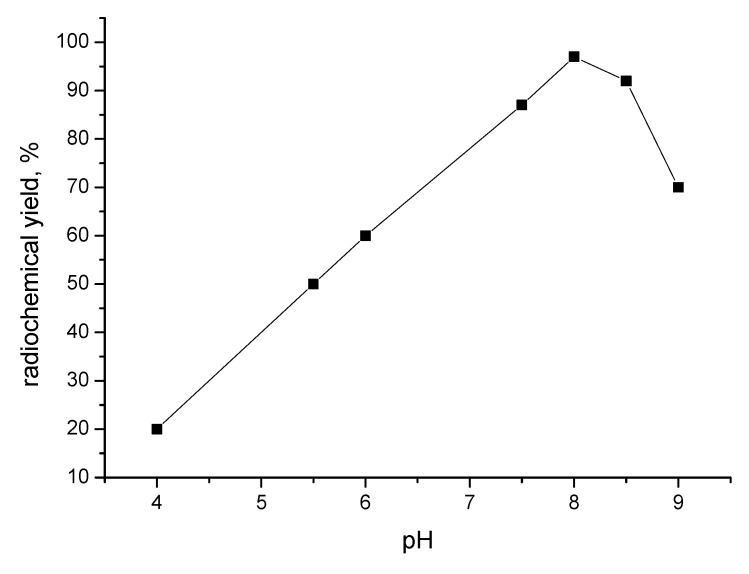
pH dependence of radiochemical yield of ^99m^Tc(CO)_3_[VP-co-AIDA] MPC (MM 15,000 Da).

The reaction yields were evaluated by HPLC on CIM columns with weak ion exchanging carboxymethyl phase in the isocratic mode. It was shown that the maximum yield is achieved at pH 8; then, a decrease in the reaction yield was observed (Figure 5). The small yield of the reaction at low pH can be explained by the protonation of the IDA carboxylic groups, which reduces their reactivity. Meanwhile, strongly alkaline media facilitate the substitution of one water molecule in ^99m^Tc(CO)_3_(H_2_O)_3_^+^ with hydroxyl ion and the formation of a weakly reactive complex ^99m^Tc(CO)_3_(H_2_O)_2_(OH).

Similar dependences were observed for samples with molecular masses equal to 9000 Da and 30,000 Da; thus, it can be concluded that the radiolabeling yield does not depend on molecular mass of the copolymer.

**Figure 6:**
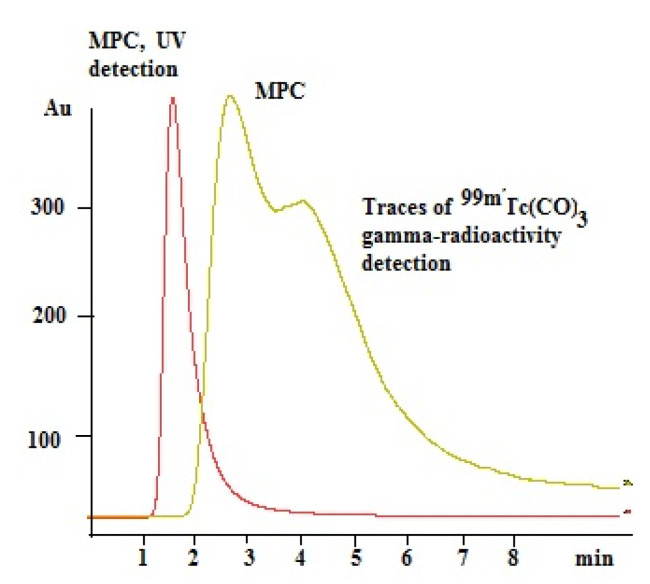
HPLC profile of the reaction mixture containing VP-co-AIDA copolymer (15,000 Da) and ^99m^Tc(CO)_3_ species (reaction mixture at pH 5.5) (CIM CM column 0.5 cm × 1 cm); 0.1% TFA/acetonitrile, 0.3 mL/min (red-UV detection at 210 nm, yellow-γ-radioactivity detection).

A typical HPLC profile of the reaction mixture is shown at Figure 6 (pH 5.5). Resolved peaks of radiolabeled polymer and starting ^99m^Tc(CO)_3_ species could be observed. The highest radiochemical yield was equal to 95–97% (pH 8) (Figure 4); this product can be used in biological studies without additional purification.

According to the HPLC data, the target ^99m^Tc(CO)_3_-VP-co-AIDA metal–polymer complexes were stable in aqueous solutions at room temperature at pH values ranging from 2 to 9 for at least 24 h.

**Figure 7:**
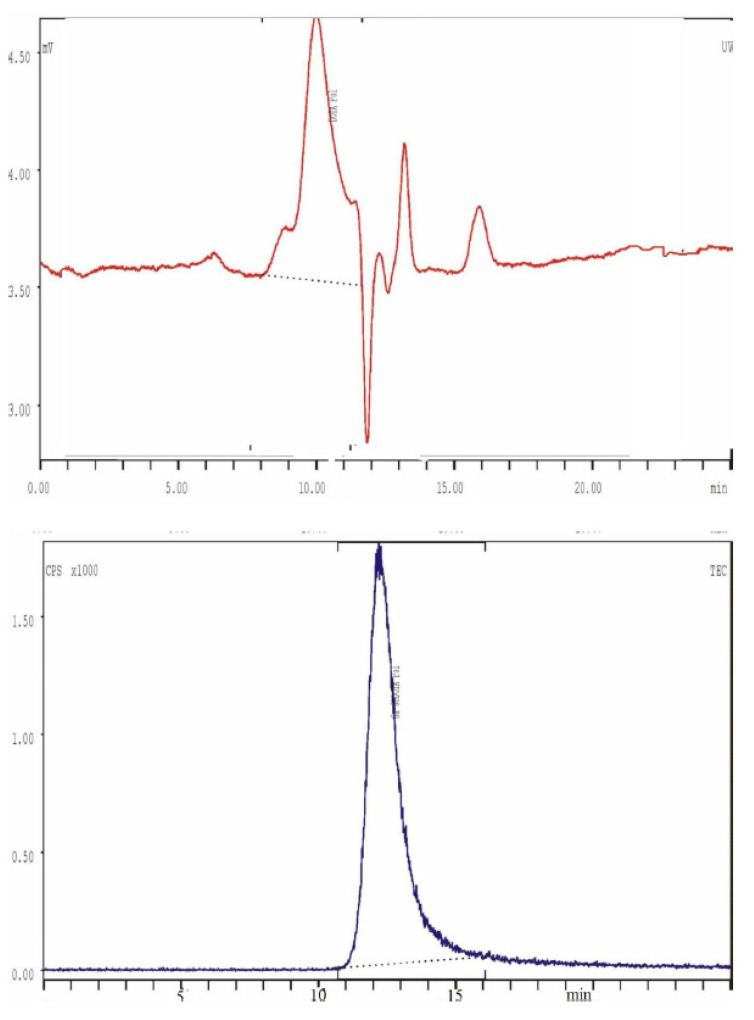
HPLC profile of ^99m^Tc(CO)_3_-VP-co-AIDA MPC (MM = 15,000 Da) in rat blood serum. Ultrahydrogel Linear SEC column (0.78 cm × 30 cm), elution 0.2 M NaCl, 0.8 mL/min. Upper chromatogram: rat serum UV detection (λ = 210 nm, RT = 9.8 min), lower chromatogram: gamma radioactivity detection (RT = 12.18 min)- ^99m^Tc(CO)_3_-VP-co-AIDA (MM = 15,000 Da) incubated in serum for 180 min.

The stability of the ^99m^Tc(CO)_3_-VP-co-AIDA complex in vitro was assessed by the incubation of the complex in the blood serum of rats for 15–180 min at 37 °C (Figure 7). The mixture was analyzed by HPLC, which made it possible to separate serum proteins (RT varying from 6.0 to 9.4 min) and the metal–polymer complex (RT 9.8 to 9.9 min). It was shown that the ^99m^Tc(CO)_3_-VP-co-AIDA metal–polymer complex is sufficiently stable for 3 h.

### Biodistribution Studies

**Figure 8:**
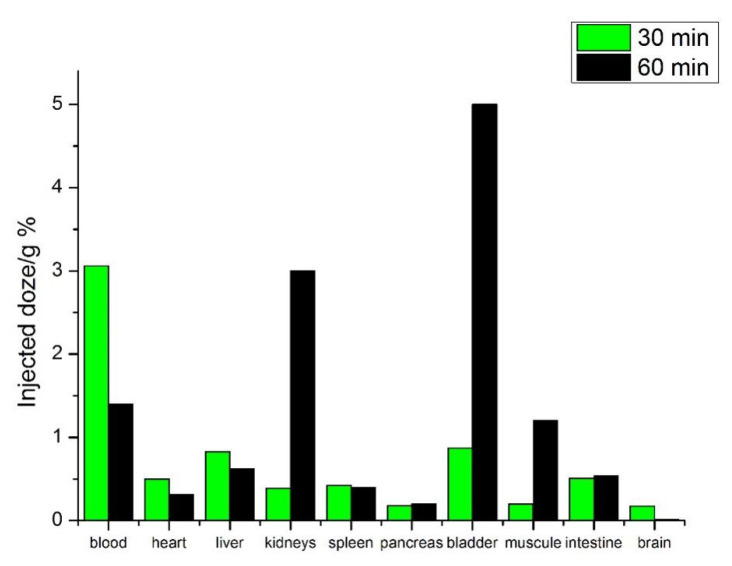
Diagram of biodistribution of ^99m^Tc(CO)_3_ -VP-AIDA (MM = 15,000 Da) in intact laboratory animals (Wistar rats).

Biodistribution of MPC ^99m^Tc(CO)_3_ -VP-AIDA (MM = 15,000 Da) in intact laboratory animals (Wistar rats) was investigated (Figure 8. It could be seen from the presented data that in the case of intact animals, the radiolabeled MPC is primarily accumulated in the bloodstream (30 min) (3.1% of the injected dose per 1 g of a tissue (ID/g (%)) and in the urinary tract (kidney (0.86%) and bladder (0.9%)). It is noteworthy that the active substance is rather rapidly (in 60 min after injection) excreted through the urinary tract (kidney (3.2%) and bladder (5.2%)). The noticeable accumulation of the radioactive substance in liver was also observed ((0.83% (30 min), (0.61% (60 min)). At the same time, the very slow accumulation of activity in muscle tissues was observed (0.21% (30 min), 1,2% (60 min).

## 4. Conclusions

Novel complexes of rhenium tricarbonyl with copolymers of N-vinylpyrrolidone and allyliminodiacetate with molecular masses suitable for the realization of the EPR effect were synthesized under mild conditions and characterized by several physico-chemical methods. The coordination of Re(CO)_3_ moiety to polymer matrix was confirmed by spectral studies. The combination of IR and ^1^H NMR spectroscopy allowed to confirm the introduction of M(CO)_3_^+^ moieties into the VP-AIDA polymeric backbone. A fast and efficient procedure for chromatographic separation of macromolecular and organometallic components using ultrashort monolith columns was proposed; the technique is suitable for fast and effective radiochemical analysis. The achieved radiochemical yields of the target MPC were sufficiently high for biological studies; their specific activity was revealed. The prepared MPCs were stable in rat blood serum for 3 h. The biodistribution of the sample of ^99m^Tc(CO)_3_ -VP-AIDA with MM 15,000 Da in laboratory animals (Wistar rats) demonstrated relatively fast clearance via urinary traction, but the growth of activity accumulation in muscle tissues makes it a promising object for the further development of novel synthetic polymeric carriers for target chemistry.

## Figures and Tables

**Table 1:** Synthetic conditions for VP-co-AA copolymers.

Copolymerization Conditions			Copolymer			
Solvent	(VP)/(AA), mol.%	Yield, %	(η), dl/g	Mη	(AA)	
					mol.%	wt.%
ethanol	50:50	20	0.08	9000	11.5	6.2
ethanol	50:50	20	0.10	15,000	11.6	6.3
ethanol	70:30	43	0.16	30,000	5.6	2.9

**Table 2:** Molecular mass distributions of VP-co-AIDA copolymers and the corresponding VP-co-AIDA-[Re(CO_3_)] metal-polymer complexes.

Mη.	*R*_T_	*M*p	*M*n	*M*w	*M*w*/M*n
VP-co-AIDA					
9000	9,39	9600	4800	10,600	2.2
15,000	8,48	14,500	11,600	26,700	2.3
30,000	7.47	28,800	18,700	45,800	2.4
VP-co-AIDA-[Re(CO_3_)]					
9000	9,32	9200	4600	10,300	2.2
15,000	8,47	14,300	11,400	26,900	2.4
30,000	7.36	29,900	18,300	46,300	2.5

*R*_T_ is the retention time; *M*_p_ is the retention time at peak maximum; *M*_n_ is the number-average molecular mass; *M*_W_ is the weight-average molecular mass; *M*_w_/*M*_n_ is the polydispersity index.

## Data Availability

The data presented in this study are available on request from the corresponding author.
